# Supplementary material for: Stimulus-responsive assembly of nonviral nucleocapsids
Source: Nat Commun. 2024 Apr 27;15:3576. doi: 10.1038/s41467-024-47808-1 (PMC11055949; doi:10.1038/s41467-024-47808-1)
Supplement: Supplementary file 1 — Supplementary information [file 41467_2024_47808_MOESM1_ESM.pdf]

Supplementary Figures and Tables for

Stimulus-responsive assembly of nonviral nucleocapsids

Mao Hori[a,b,†], Angela Steinauer[a,c,†], Stephan Tetter[a], Jamiro Hälg[a], Eva-Maria Manz[a], Donald Hilvert[a,*]

[a] *Laboratory of Organic Chemistry, ETH Zürich, 8093 Zürich, Switzerland*

[b] *Present address: Present address: Institute of Biomaterials and Bioengineering, Tokyo Medical and Dental University, 2-3-10 Kanda-Surugadai, Chiyoda-ku, Tokyo 101-0062, Japan*

[c] *Present address: École Polytechnique Fédérale de Lausanne (EPFL), SB ISIC LIBN, 1015 Lausanne, Switzerland*

[d] *Present address: MRC Laboratory of Molecular Biology, Francis Crick Avenue, CB21LA, Cambridge, UK*

[†] These authors contributed equally to this work.

[*] *Correspondence to: hilvert@org.chem.ethz.ch*

The PDF file includes:

Supplementary Fig. 1. ESI-MS spectrum of NC-4 monomer 2

Supplementary Fig. 2. NC-4 monomers form insoluble aggregates without RNAs 3

Supplementary Fig. 3. In vitro nucleocapsid assembly components visualized by native-AGE ... 4

Supplementary Fig. 4. Optimization of the salt concentration for in vitro assembly of the nucleocapsids 5

Supplementary Fig. 5. Optimizing the MBP-NC-4 to NC-4 RNA ratio 6

Supplementary Fig. 6. TEM image of the in vitro assembled NC-4 7

Supplementary Fig. 7. Gold-standard Fourier-Shell correlation curve 8

Supplementary Fig. 8. Encapsidation of different RNA cargo 9

Supplementary Table 1. Protein sequences of the NC-4 and MBP-NC-4 10

Supplementary Table 2. mRNA sequences 11

Supplementary Table 3. CryoEM data collection and processing 13

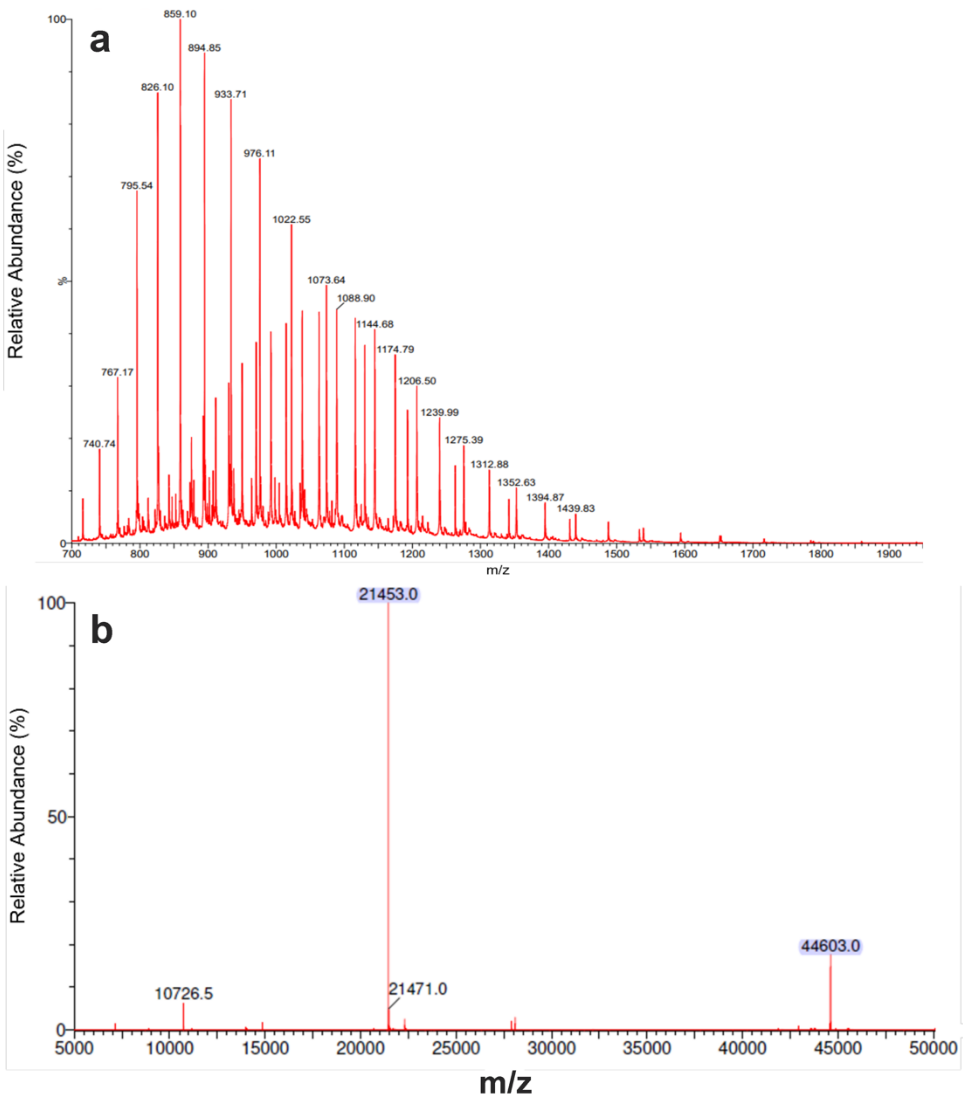

Supplementary Fig. 1 | ESI-MS analysis of the NC-4 monomer. a, non-deconvoluted ESI-MS spectrum of the mixture obtained by treating the SEC-purified MBP-NC-4 fusion protein with 0.1 U/µL TEV protease, followed by desalting the sample using a C18 ZipTip column (Millipore, USA), showing the presence of both fragment proteins. Measurement and analysis details are described in the experimental methods. **b,** deconvoluted ESI-MS spectrum of the NC-4 monomer in the mixture. The measured mass, 21453.00 Da, corresponds to the calculated average mass of the NC-4 monomer, 21453.23 Da.

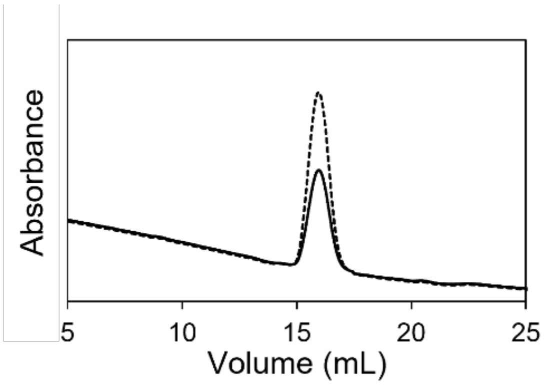

Supplementary Fig. 2 | NC-4 monomers form insoluble aggregates without RNAs. SEC trace of MBP-NC-4 after TEV protease treatment at room temperature overnight showing the absorbance at 260 nm (solid line) and 280 nm (dashed line). The sample was filtered through a 0.2 µm syringe filter before loading onto a Superdex 200 increase 10/300 GL column (GE Healthcare, Chicago, IL, USA). The elution volume (~16 mL) corresponds to the molecular mass of MBP (~44 kDa); no fusion protein is observed, indicating complete cleavage by TEV protease. Neither NC4 monomers nor higher-order assemblies are observed in the absence of RNA, consistent with their aggregation and removal in the filtration step. This experiment was independently repeated three times with similar results.

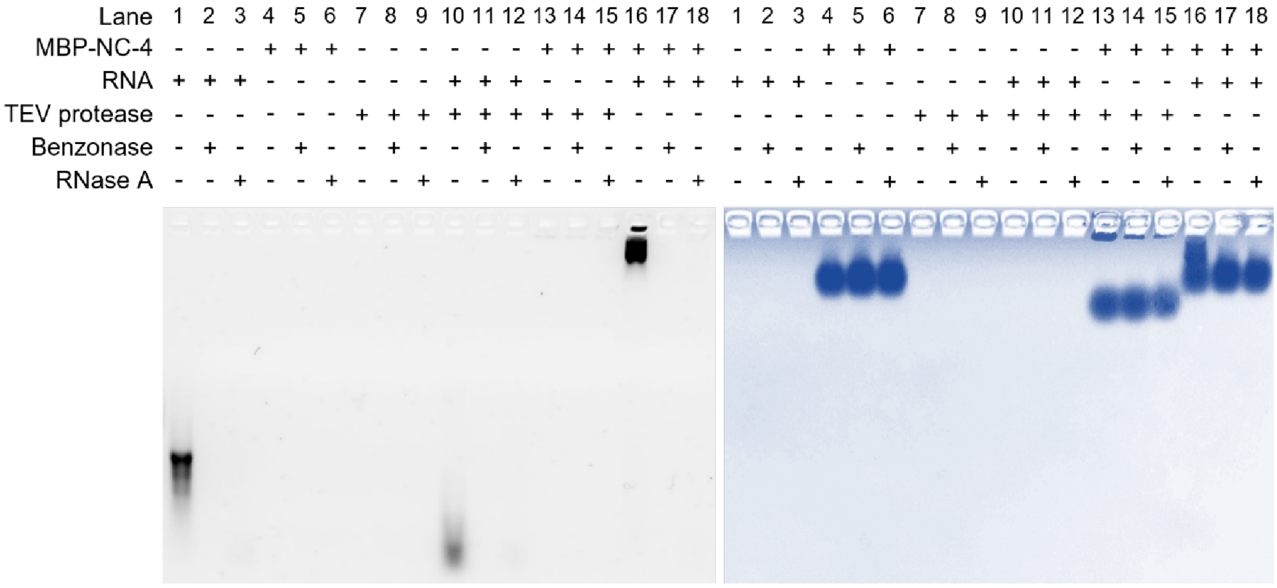

Supplementary Fig. 3 | In vitro nucleocapsid assembly components visualized by native-AGE. Native-AGE images of MBP-NC-4, NC-4 RNA, TEV protease, benzonase, RNAse A, and their mixtures stained either with GelRed (left) or Coomassie Brilliant Blue (right). Each component was incubated individually or in combination with one or two other components for 1 h at room temperature prior to loading onto the gel. Final concentrations of each component are as follows; MBP-NC-4: 48 μM, NC-4 RNA: 0.5 μM, TEV protease: 0.1 U/μL, benzonase: 2.5 U/μL, RNase A: 10 μg/mL. This experiment was repeated on three independent sample batches with similar results.

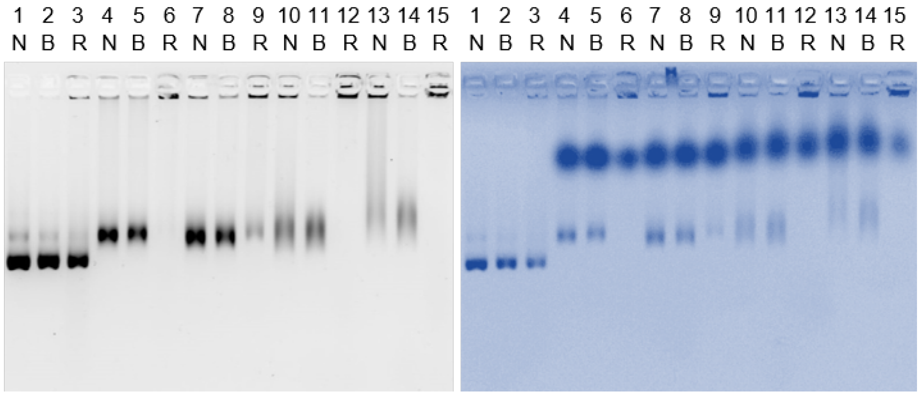

Supplementary Fig. 4 | Optimization of the salt concentration for in vitro assembly of the nucleocapsids. Native-AGE images of *in vivo* (1-3) and *in vitro* assembled NC-4 prepared in phosphate buffer containing 150 (4-6), 300 (7-9), 600 (10-12), 800 (13-15) mM NaCl before (N: non-treated) and after benzonase (B) or RNase A (R) treatment. The gels were stained with either GelRed (left) or Coomassie Brilliant Blue (right). This experiment was repeated on three independent sample batches with similar results.

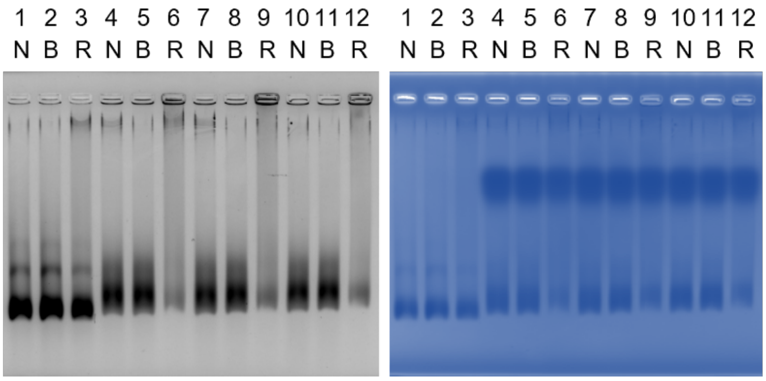

Supplementary Fig. 5 | Optimizing the MBP-NC-4 to NC-4 RNA ratio. Native-AGE images of *in vivo* (1-3) and *in vitro* assembled NC-4 prepared at the molar ratio of MBP-NC-4 to NC-4 RNA = 96:1 and then a second addition of 26 (4-6), 20 (7-9) and 13 (10-12) eq. of MBP-NC-4 followed by 24 h incubation at room temperature. (left: GelRed, right Coomassie Brilliant Blue, N: no treatment, B: benzonase treatment, R: RNase A treatment). This experiment was repeated on three independent sample batches with similar results.

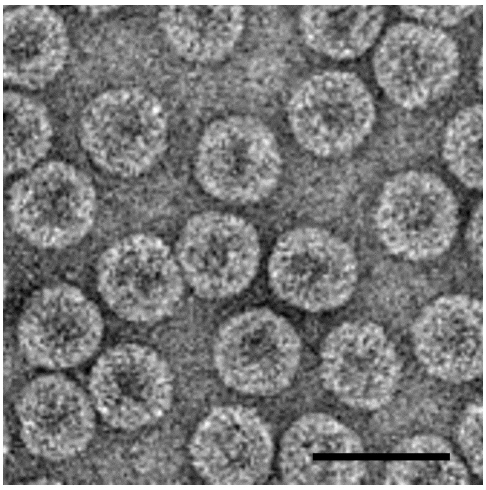

Supplementary Fig. 6 | TEM image of the in vitro assembled NC-4. The in vitro assembled NC-4 was prepared by the optimized protocol and purified by anion-exchange chromatography. The purified sample was used for TEM measurements after negative staining with 2% (w/v) uranyl acetate solution. The grid was prepared and visualized as described in the section on "Negative-stain transmission electron microscopy (TEM)" (scale bar: 50 nm). This experiment was repeated on three independent sample batches with similar results.

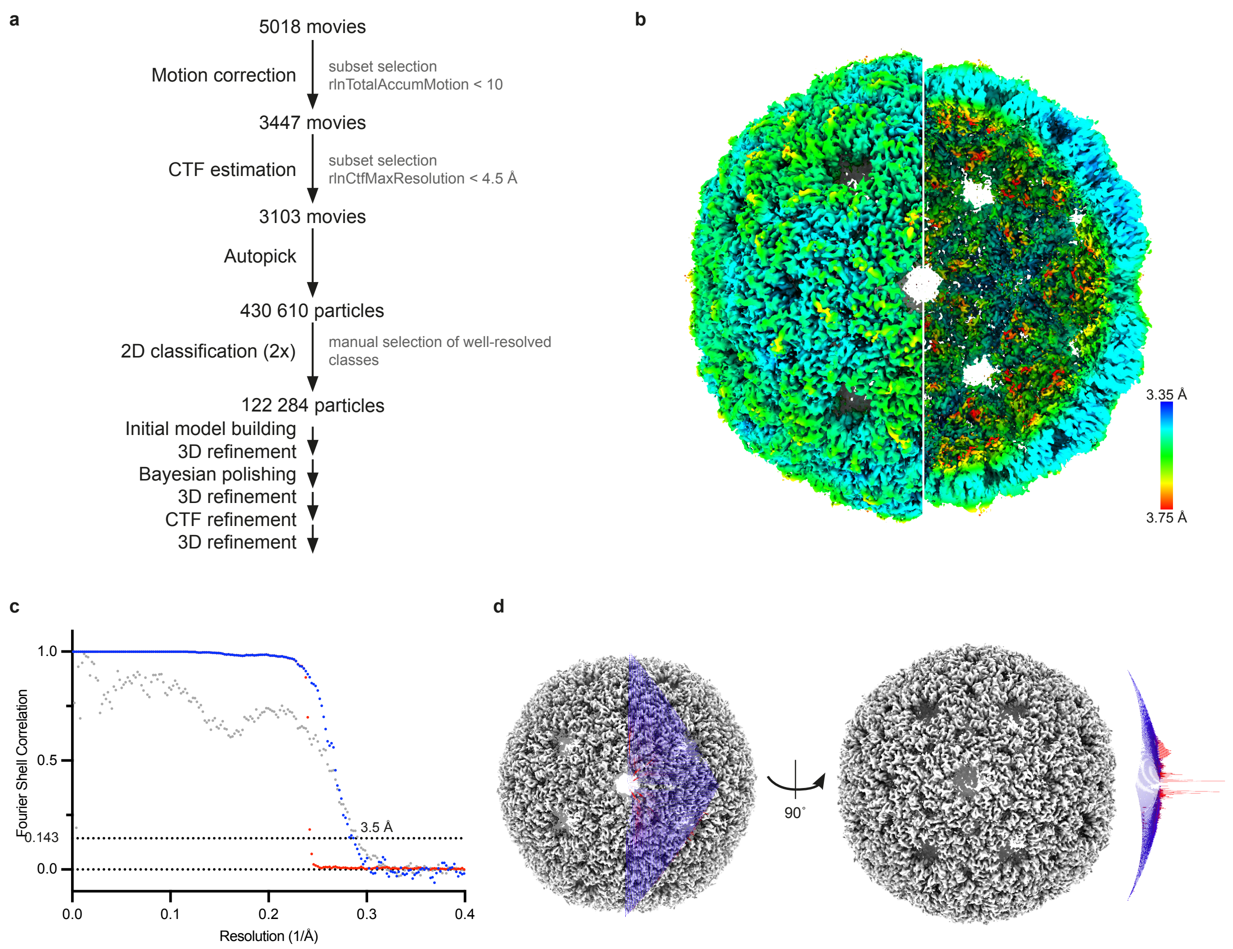

Supplementary Fig. 7 | CryoEM data processing and analysis. a, Diagram of the data processing workflow. 5018 movies were collected on a TFS Glacios microscope. Movies were motion corrected and the contrast transfer function (CTF) was estimated; the best micrographs were selected at each step as indicated. After 2D classification of about 1000 manually picked particles, reference-based autopicking was performed. The best-looking classes after two rounds of 2D classification were reextracted at full resolution. Initial model building was performed with imposed icosahedral symmetry. Refined particles were subjected to particle polishing and CTF refinement. 3D classification of the final refined map did not provide subsets leading to improved resolution or alternative conformations. **b,** The postprocessed and masked map is depicted coloured by local resolution. The left half shows the external view of the protein cage, while the right half shows a centrally sliced view. **c,** Fourier shell correlation between the two independently refined half-maps (blue), with the resolution indicated at 0.143. The model versus map correlation (grey) shows good overlap between the in vitro-assembled capsids and the atomic model of in vivo-assembled capsids (PDB-ID 7A4J), supporting that both assembly paths yield the same structure. The correlation between phase-randomized masked maps (red) shows that an appropriately soft mask was used for postprocessing. **d,** Angular distribution plots of particles included in the final 3D reconstruction viewed from two perspectives. The number of particles with respective orientations are represented by length and coloured cylinders, ranging from blue to red. In the centre, the cryo-EM map is shown in grey.

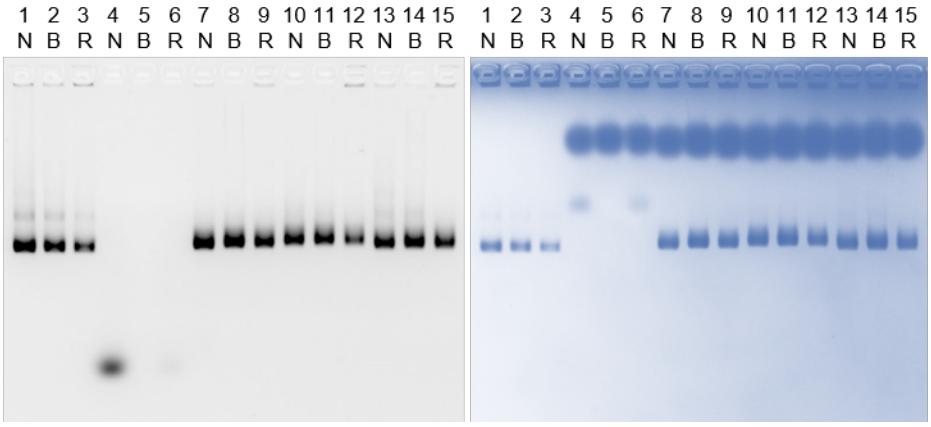

Supplementary Fig. 8 | Encapsidation of different RNA cargo. Native-AGE images of *in vivo* (1-3) and *in vitro* assembled NC-4 prepared using the RNAs encoding boxB tag (4-6), HIV protease (7-9), BoxBr-HIV protease-BoxBr (10-12), and BoxBr-NC-4 monomer-HIV protease-BoxBr (13-15) (left: GelRed, right Coomassie Brilliant Blue, N: no treatment, B: benzonase treatment, R: RNase A treatment). This experiment was repeated on three independent sample batches with similar results.

Supplementary Table 1 Protein sequences of NC-4 and MBP-NC-4 (red = NC-4).

Protein name	Amino acid sequence
NC-4	MGSGNARTRRRERRAEKQAQWKAANAGAGAGAMATPHFDYNASVVSKGLANLSLELRKPVSFDIITADTLEQAIERAGTKHGNKGWEAALSAIEMANLYKSLRGTEHHHHLHGSSIEIYEGKLTAEGLRFGIVASRFNHTLVDRLVEGAIDCIVRHGGRGEDITLVRVPGAWEIPVAADELARKEDIDAVIAFGDLIRG
MBP-NC-4	MRGSHHHHHHGSKIEEGKLVIWINGDKGYNGLAEVGKKFEKDTGIKVTVEHPDKLEEKFPQVAATGDGPDIIFWAHDRFGGYAQSGLLAEITPDKAFQDKLYPFTWDAVRYNGKLIAYPIAVEALSLIYNKDLLPNPPKTWEEIPALDKELKAKGKSALMFNLQEPYFTWPLIAADGGYAFKYENGKYDIKDVGVDNAGAKAGLTFLVDLIKNKHMNADTDYSIAEAAFNKGETAMTINGPWAWSNIDTSKVNYGVTVLPTFKGQPSKPFVGVLSAGINAASPNKELAKEFLENYLLTDEGLEAVNKDKPLGAVALKSYEEELAKDPRIAATMENAQKGEIMPNIPQMSAFWYAVRTAVINAASGRQTVDEALKDAQTNSSSNNNNNNNNNLGIEGTTENLYFQGSGNARTRRRERRAEKQAQWKAANAGAGAGAMATPHFDYNASVVSKGLANLSLELRKPVSFDIITADTLEQAIERAGTKHGNKGWEAALSAIEMANLYKSLRGTEHHHHLHGSSIEIYEGKLTAEGLRFGIVASRFNHTLVDRLVEGAIDCIVRHGGRGEDITLVRVPGAWEIPVAADELARKEDIDAVIAFGDLIRG

Supplementary Table 2 mRNA sequences (green = BoxBr tags, red = NC-4 monomer, blue = HIV protease).

mRNA name	mRNA sequence
BoxBr-NC-4 monomer-BoxBr	GGGGAAUUGUGAGCGGAUAACAAUUCCCCUCUAGAGGGGAGACG<br>GUCGGGUCCGGGCCCUGAAGAAGGGCCCGUCGAGUAGAGUGUGG<br>GCUCCCCGAAAUAAUUUUGUUUAACUUUAAGAAGGAGAUAUACA<br>UAUGGGAAACGCGAGAACGCGACGCCGCGAACGCCGCGCUGAGAA<br>ACAGGCACAGUGGAAAGCCGCCAACGCUGGAGCUGGAGCAGGUGC<br>AAUGGCGACGCCACAUUUCGAUUAUAACGCCUCUGUAGUUUCAA<br>AAGGCCUCGCGAACCUUUCAUUAGAACUACGUAAACCUGUCUCCU<br>UCGACAUUAUUACAGCUGACACCUUGGAACAGGCUAUCGAGCGCG<br>CCGGCACAAAACACGGCAACAAAGGUUGGGAAGCAGCGCUUUCUG<br>CCAUUGAAAUGGCAAACUUAUACAAGUCUCUCCGAGGUACCGAGC<br>ACCAUCACCAUCUUCACGGGAGCUCGAUUGAAAUCUACGAAGGUA<br>AACUAACUGCCGAAGGCCUUCGUUUCGGUAUCGUAGCAUCACGUU<br>UUAAUCAUACCCUUGUCGAUCGUCUGGUGGAGGGAGCAAUUGAU<br>UGCAUAGUCCGUCAUGGCGGCCGUGGAGAAGACAUUACUCUGGU<br>UCGUGUUCCAGGCGCAUGGGAAAUACCGGUUGCUGCGGAUGAAC<br>UGGCGCGUAAAGAGGACAUUGACGCUGUUAUCGCGUUUGGCGAU<br>CUCAUCAGAGGCUAACUCGAGUAAGCGGCGAGGGGAGACGGUCG<br>GGUCCGGGCCCUGAAGAAGGGCCCGUCGAGUAGAGUGUGGGCUCC<br>CCGCUGAGCAAUAACUAGCAUAACCCCUUGGGGCCUCUAAACGGG<br>UCUUGAGGGGUUUUUUG
boxB	GCCCUGAAGAAGGGC
HIV protease	GGGGAAUUGUGAGCGGAUAACAAUUCCCCUCUAGAAAUAAUUUU<br>GUUUAACUUUAAGAAGGAGAUAUACAUAUGCCUCAGAUCACUCU<br>UUGGCAACGACCCCUCGUCACAAUAAAGAUAGGGGGGCAACUAA<br>AGGAAGCUCUAUUAGAUACAGGAGCAGAUGAUACAGUAUUAGAA<br>GAAAUGAGUUUGCCAGGAAGAUGGAAACCAAAAAUGAUAGGGGG<br>AAUUGGAGGUUUUAUCAAAGUAAGACAGUAUGAUCAGAUACUCA<br>UAGAAAUCUGUGGACAUAAAGCUAUAGGUACAGUAUUAGUAGGA<br>CCUACACCUGUCAACAUAAUUGGAAGAAAUCUGUUGACUCAGAU<br>UGGUUGCACUUUAAAUUUUUAAACUAGUCAGCUGAUCCGGCUGC<br>UAACAAAGCCCGAAAGGAAGCUGAGUUGGCUGCUGCCACCGCUGA<br>GCAAUAACUAGCAUAACCCCUUGGGGCCUCUAAACGGGUCUUGAG<br>GGGUUUUUUG

BoxBr-HIV protease-BoxBr	GGGGAAUUGUGAGCGGAUAACAAUUCCCCUCUAGAGGGGAGACG<br>GUCGGGUCCGGGCCCUGAAGAAGGGCCCGUCGAGUAGAGUGUGG<br>GCUCCCCGAAAUAAUUUUGUUUAACUUUAAGAAGGAGAUAUACA<br>UAUGCCUCAGAUCACUCUUUGGCAACGACCCCUCGUCACAAUAAA<br>GAUAGGGGGGCAACUAAAGGAAGCUCUAUUAGAUACAGGAGCAG<br>AUGAUACAGUAUUAGAAGAAAUGAGUUUGCCAGGAAGAUGGAAA<br>CCAAAAAUGAUAGGGGGAAUUGGAGGUUUUAUCAAAGUAAGACA<br>GUAUGAUCAGAUACUCAUAGAAAUCUGUGGACAUAAAGCUAUAG<br>GUACAGUAUUAGUAGGACCUACACCUGUCAACAUAAUUGGAAGA<br>AAUCUGUUGACUCAGAUUGGUUGCACUUUAAAUUUUUAAACUAG<br>UCAGCUGAUCCGGGGAGACGGUCGGGUCCGGGCCCUGAAGAAGG<br>GCCCGUCGAGUAGAGUGUGGGCUCCCCGCUGAGCAAUAACUAGCA<br>UAACCCCUUGGGGCCUCUAAACGGGUCUUGAGGGGUUUUUUG
BoxBr-NC-4 monomer-HIV protease-BoxBr	GGGGAAUUGUGAGCGGAUAACAAUUCCCCUCUAGAGGGGAGACG<br>GUCGGGUCCGGGCCCUGAAGAAGGGCCCGUCGAGUAGAGUGUGG<br>GCUCCCCGAAAUAAUUUUGUUUAACUUUAAGAAGGAGAUAUACA<br>UAUGGGAAACGCGAGAACGCGACGCCGCGAACGCCGCGCUGAGAA<br>ACAGGCACAGUGGAAAGCCGCCAACGCUGGAGCUGGAGCAGGUGC<br>AAUGGCGACGCCACAUUUCGAUUAUAACGCCUCUGUAGUUUCAA<br>AAGGCCUCGCGAACCUUUCAUUAGAACUACGUAAACCUGUCUCCU<br>UCGACAUUAUUACAGCUGACACCUUGGAACAGGCUAUCGAGCGCG<br>CCGGCACAAAACACGGCAACAAAGGUUGGGAAGCAGCGCUUUCUG<br>CCAUUGAAAUGGCAAACUUAUACAAGUCUCUCCGAGGUACCGAGC<br>ACCAUCACCAUCUUCACGGGAGCUCGAUUGAAAUCUACGAAGGUA<br>AACUAACUGCCGAAGGCCUUCGUUUCGGUAUCGUAGCAUCACGUU<br>UUAAUCAUACCCUUGUCGAUCGUCUGGUGGAGGGAGCAAUUGAU<br>UGCAUAGUCCGUCAUGGCGGCCGUGGAGAAGACAUUACUCUGGU<br>UCGUGUUCCAGGCGCAUGGGAAAUACCGGUUGCUGCGGAUGAAC<br>UGGCGCGUAAAGAGGACAUUGACGCUGUUAUCGCGUUUGGCGAU<br>CUCAUCAGAGGCUAACUCGAGCAUAUGCCUCAGAUCACUCUUUGG<br>CAACGACCCCUCGUCACAAUAAAGAUAGGGGGGCAACUAAAGGA<br>AGCUCUAUUAGAUACAGGAGCAGAUGAUACAGUAUUAGAAGAAA<br>UGAGUUUGCCAGGAAGAUGGAAACCAAAAAUGAUAGGGGGAAUU<br>GGAGGUUUUAUCAAAGUAAGACAGUAUGAUCAGAUACUCAUAGA<br>AAUCUGUGGACAUAAAGCUAUAGGUACAGUAUUAGUAGGACCUA<br>CACCUGUCAACAUAAUUGGAAGAAAUCUGUUGACUCAGAUUGGU<br>UGCACUUUAAAUUUUUAAACUAGUCAGCUGAUCCGGGGAGACGG<br>UCGGGUCCGGGCCCUGAAGAAGGGCCCGUCGAGUAGAGUGUGGG<br>CUCCCCGCUGAGCAAUAACUAGCAUAACCCCUUGGGGCCUCUAAA<br>CGGGUCUUGAGGGGUUUUUUG

Supplementary Table 3 CryoEM data collection and processing.

EMDB map entry	16696
Microscope model	TFS Glacios
Detector model	Falcon III
Number of Micrographs collected	5 019
Magnification	120 000
Voltage (kV)	200
Electron dose (e-/A2)	40
Pixel Size (Å)	1.24
Defocus range (µm)	-0.8 to -2.2
Symmetry imposed	I
Number of Micrographs used	3 103
Initial particle images	430 610
Final particle images	122 284
Resolution (Å) (at FSC = 0.143)	3.5
Map sharpening B-factor (Å2)	-245

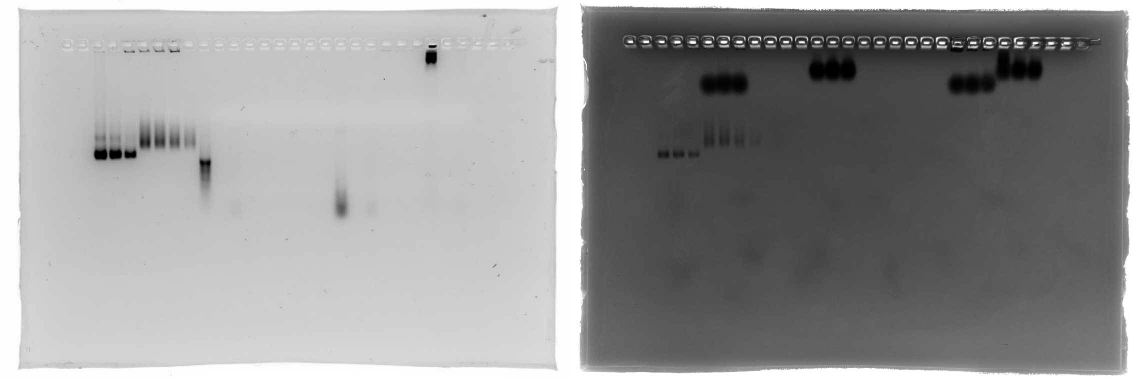

Uncropped scans of the gels presented in Supplementary Fig. 3

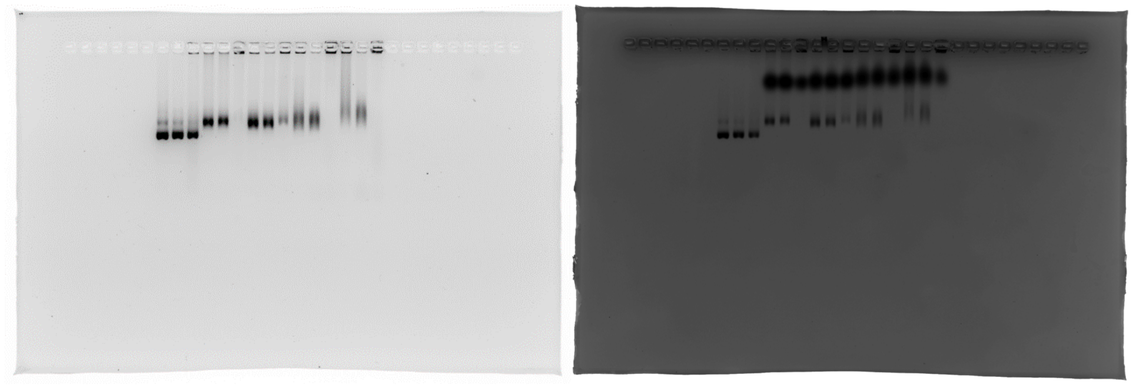

Uncropped scans of the gels presented in Supplementary Fig. 4

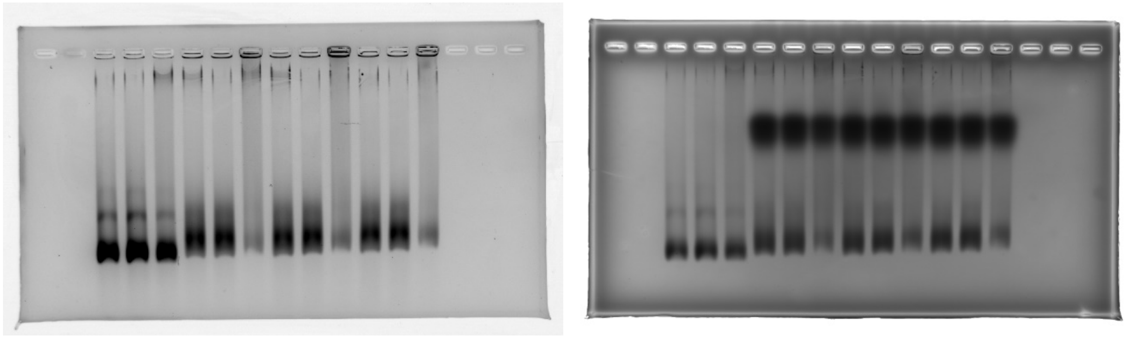

Uncropped scans of the gels presented in Supplementary Fig. 5

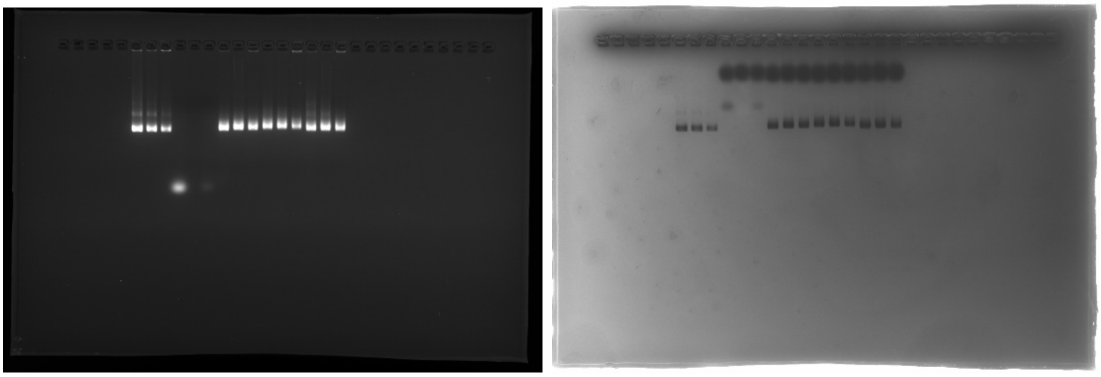

Uncropped scans of the gels presented in Supplementary Fig. 8.
